# Development of a Bayesian Estimator for Audio-Visual Integration: A Neurocomputational Study

**DOI:** 10.3389/fncom.2017.00089

**Published:** 2017-10-04

**Authors:** Mauro Ursino, Andrea Crisafulli, Giuseppe di Pellegrino, Elisa Magosso, Cristiano Cuppini

**Affiliations:** ^1^Department of Electrical, Electronic and Information Engineering, University of Bologna, Bologna, Italy; ^2^Department of Psychology, University of Bologna, Bologna, Italy

**Keywords:** neural networks, prior probability, multisensory integration, perception bias, ventriloquism

## Abstract

The brain integrates information from different sensory modalities to generate a coherent and accurate percept of external events. Several experimental studies suggest that this integration follows the principle of Bayesian estimate. However, the neural mechanisms responsible for this behavior, and its development in a multisensory environment, are still insufficiently understood. We recently presented a neural network model of audio-visual integration (Neural Computation, 2017) to investigate how a Bayesian estimator can spontaneously develop from the statistics of external stimuli. Model assumes the presence of two unimodal areas (auditory and visual) topologically organized. Neurons in each area receive an input from the external environment, computed as the inner product of the sensory-specific stimulus and the receptive field synapses, and a cross-modal input from neurons of the other modality. Based on sensory experience, synapses were trained via Hebbian potentiation and a decay term. Aim of this work is to improve the previous model, including a more realistic distribution of visual stimuli: visual stimuli have a higher spatial accuracy at the central azimuthal coordinate and a lower accuracy at the periphery. Moreover, their prior probability is higher at the center, and decreases toward the periphery. Simulations show that, after training, the receptive fields of visual and auditory neurons shrink to reproduce the accuracy of the input (both at the center and at the periphery in the visual case), thus realizing the likelihood estimate of unimodal spatial position. Moreover, the preferred positions of visual neurons contract toward the center, thus encoding the prior probability of the visual input. Finally, a prior probability of the co-occurrence of audio-visual stimuli is encoded in the cross-modal synapses. The model is able to simulate the main properties of a Bayesian estimator and to reproduce behavioral data in all conditions examined. In particular, in unisensory conditions the visual estimates exhibit a bias toward the fovea, which increases with the level of noise. In cross modal conditions, the *SD* of the estimates decreases when using congruent audio-visual stimuli, and a ventriloquism effect becomes evident in case of spatially disparate stimuli. Moreover, the ventriloquism decreases with the eccentricity.

## Introduction

In daily life, we constantly localize objects in space, by merging information coming from different sensory modalities, with different spatial and temporal reliability and corrupted by noise. This capacity to provide an optimal localization, by minimizing errors, is crucial for animal survival and for almost all motor and cognitive problems involving interactions with the environment.

A wide amount of literature, both theoretical (Patton and Anastasio, 2003; Pouget et al., 2003, 2013; Colonius and Diederich, 2004; Ma et al., 2006; Ma and Rahmati, 2013; Rich et al., 2015), and experimental (Shams et al., 2000, 2005a; Alais and Burr, 2004; Körding et al., 2007; Gu et al., 2008; Fetsch et al., 2009, 2012; Fischer and Peña, 2011; Cazettes et al., 2016), suggests that the brain uses a Bayesian approach to combine stimuli in order to estimate their spatial localization. According to the Bayes rule, an optimal estimate (i.e., one that minimizes the probability of error) can be achieved by computing the maximal posterior probability. The latter, in turn, depends on two different pieces of information: The *likelihood probability* of the stimulus, which captures the process of stimulus generation (for instance, the effect of noise, or the stimulus spatial tuning), and the *prior probability*, which summarizes past experience on the parameter to be estimated (for instance, how frequently the stimulus occurred at a given position, how cross-modal stimuli are spatially linked).

Mathematical equations, based on the Bayes theorem, provided accurate reproduction of behavioral data in a variety of conditions, including the ventriloquism effect (Alais and Burr, 2004; Ursino et al., 2017), the fission effect (Shams et al., 2005b), the causal inference problem (Wozny et al., 2010). See Ursino et al. (2014) for a review.

Two important problems, however, are still a matter of debate in cognitive neuroscience: Does the brain implement the Bayesian estimate via biological neural circuits? How can the likelihood and prior probabilities be extracted from the stimulus and from the previous experience, and encoded in the topological structure of a neural net?

The last question is strictly related with the problem of how a neural net develops during the early period of life. Indeed, if spatial estimate follows the principles of Bayesian inference, a training period is necessary to infer the nature of the generative process and, above all, to construct a prior from previous experience. Accordingly, various experimental (Wallace and Stein, 1997; Froemke and Jones, 2011; Pecka et al., 2014) and behavioral (Birch et al., 1983; Gori et al., 2008; Nardini et al., 2008; Johnson, 2010; Aslin and Newport, 2012) results suggest that spatial localization capabilities, both in the cortex and in the superior colliculus, are not fully present at birth, but progressively develop under the pressure of multisensory environment.

In order to shed light onto the previous questions, several authors used a “neural population coding” approach (Deneve et al., 1999; Pouget et al., 2003; Ma et al., 2006; Ma and Rahmati, 2013). In this approach, information on the probability distributions is represented by the activity of a population of neurons, which code for the attributes of the input stimuli (for instance position), but without the use of learning rules. A suitable metrics is then used to extract the hidden information from the ensemble activity, i.e., to infer the estimation from the probability distributions.

Despite the previous important contributions, however, some aspects of spatial inference in the brain still deserve further theoretical analysis. In particular, we wish to focus attention on two major problems. First, learning in a neural net model is realized via synapse adjustments. Hence, which learning rule can be used by the brain to encode probabilities within a population of neurons, reflecting previous experience and the environment statistics, and which topology of synapses realizes this coding in a proper way? In particular, we are not aware of previous neural network models that use learning rules to code probabilities, but synapses are assigned a priori to reflect probabilities. Second, how can the likelihood probabilities and the prior probability be merged together within the neural population model, to automatically compute the posterior-probability required for Bayesian estimate?

To address these critical questions, in a recent paper (Ursino et al., 2017), we proposed a neural network model, consisting of two interconnected chains of unisensory neurons (let us assume, in this paper, visual and auditory, although similar ideas can be used to deal with other multisensory combinations, for instance visuo-tactile). Using a realistic learning rule (i.e., a Hebbian reinforcement with a forgetting factor) we demonstrated that the likelihood probabilities (visual and auditory, respectively) are stored in the receptive fields (RFs) of the individual neurons, while the prior probability of the co-occurrence of the stimuli (i.e., audio-visual spatial proximity) is stored in the cross-modal synapses linking the two areas. After training, the network is able to perform a maximum-likelihood estimation of the spatial position in unisensory conditions, and a near-optimal Bayesian estimation of the auditory and visual positions in cross-modal conditions. In particular, in the presence of two spatially proximal (but not-coincident) audio-visual stimuli, the model simulates the ventriloquism illusion (i.e., a shift of the auditory estimate toward the visual position) predicting an auditory perception bias as large as 8–10 deg, but quite a negligible visual perception bias, in agreement both with behavioral data (Bertelson and Radeau, 1981; Hairston et al., 2003; Wallace et al., 2004) and with the Bayesian inference.

Compared to biological reality, however, the previous paper introduced two important simplifications. First, we assumed that the spatial accuracy of the stimulus is independent of the azimuthal coordinate. Hence, during training, we used visual (auditory) stimuli with a fixed spatial resolution at all positions from 0 to 180 deg (but with the visual stimuli much more accurate than the auditory ones). Conversely, biologically data show that visual acuity is much better near the fovea, and progressively decreases in the semi-peripheral visual field (Kerr, 1971; Johnson and Leibowitz, 1979; Ransom-Hogg and Spillmann, 1980; Oehler, 1985; Strasburger et al., 2011). Second, the prior probability of the unisensory stimuli was independent of their position. In other words, we assumed that visual (auditory) stimuli occur with the same probability at all points of the spatial field. Conversely, in everyday experience, visual inputs are not uniformly distributed. Indeed, humans tend to center sight on stimuli, which leads to a greater probability of having a visual input near the fovea than in the periphery (Ludwig et al., 2014).

Aim of the present work is to improve the previous model, to account for the spatial dependence of the visual stimuli. Accordingly, we trained the network using visual stimuli with higher accuracy and higher probability in the center than in periphery. The first amendment is reflected in the likelihood probability of the visual stimuli, the second in the prior probability. Thus, both aspects significantly affect the Bayesian inference, and both require a proper synaptic change, that was not accomplished in the previous model version. In particular, it is worth noting that now the prior probability must incorporate two aspects: the non-uniform probability of the unisensory input (more frequent in certain spatial regions than in others) and the regular spatial proximity of the audio-visual stimuli.

The following aspects are then analyzed via model simulations: is the network able to encode a non-uniform likelihood (i.e., one that varies with the azimuth)? How and where are two different aspects of the prior probabilities coded in the network? Can the network, after training, produce a near-optimal Bayesian estimate, both in unisensory and cross-modal conditions?

According to the results, we claim that most aspects of these questions are satisfactorily addressed with the proposed model, thus representing a significant step toward Bayesian development in biologically inspired neural nets. Furthermore, we compared the model results with behavioral data present in literature. Similarity between human behavior and the model's results suggest that similar processes could be present in the human brain and in the proposed model.

## Materials and methods

### Qualitative model description

**Figure 1:**
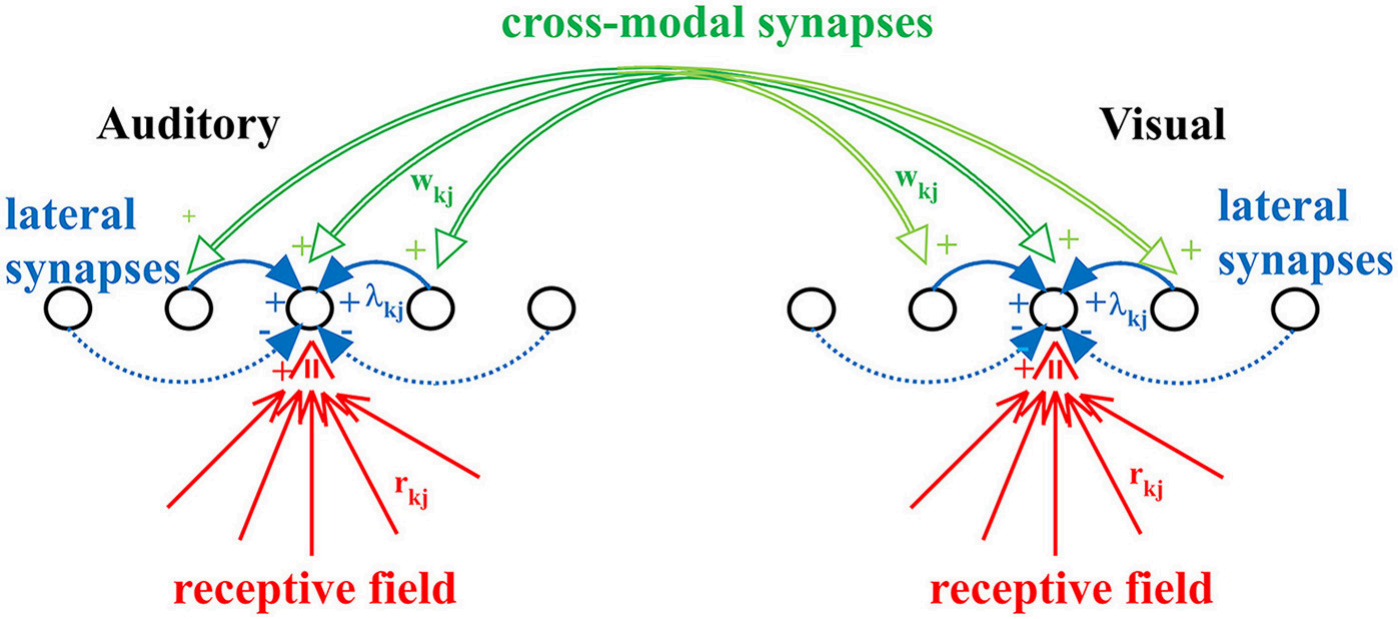
The neural network used in the present work. Each neuron accomplishes the scalar product of the external stimulus and its receptive field (r_kj_), but also receives lateral synapses (λ_kj_) from other neurons of the same modality, and cross-modal synapses (w_kj_) from neurons of the other modality. Synapses r_kj_ and w_kj_ are trained with the adopted learning rule.

The model includes two chains of unisensory neurons (one auditory and one visual) topologically organized (see Figure 1). Each neuron codes for a different portion of space, although this position can be modified by experience (see below). The activity of each neuron is simulated by means of a static sigmoidal relationship and a first-order dynamics, with time constant τ. According to the sigmoid relationship, the neuron exhibits no appreciable activity when it receives negligible input (below a given threshold) and maximal saturation activity in case of high excitatory input. In this model, the upper saturation is assumed equal to 1, i.e., all activities are normalized. The time constant describes the time required for the neuron to integrate its input and produce the response. Finally, each neuron receives lateral synapses from other elements within the same region, and cross-modal connections from neurons belonging to the other chain.

Therefore, each neuron, both in the acoustic and in the visual chain, receives three kinds of inputs:

- The external input of its specific modality (i.e., the auditory input for neurons in the auditory chain and the visual input for those in the visual one) which is spatially filtered through the neuron's receptive field. In the initial (pre-training) configuration, all neurons have the same receptive field, having identical shape characterized by large width. This is realized with a Gaussian function with *SD* = 30 deg. Moreover, we assume that the center of the receptive fields before training is uniformly distributed in space, reflecting the absence of any prior information. In this model we use 180 neurons for each layer, coding for the overall azimuthal coordinates. Hence, the initial position of the center of RFs for two consecutive neurons differs by 1 deg. An important new aspect of this work, not incorporated in the previous version, is that the preferred position of each neuron is not fixed, but can shift as a result of the sensory training, to incorporate the statistics of the unisensory inputs. To this end, the preferred position is computed as the barycenter of its RF. In particular, after training (see section Results) the RFs of all neuron shrink (to reflect the likelihood of the external input) and their position moves (to reflect the prior probability of the external input).
- A lateral input from other neurons in the same layer through the lateral synapses. These connections have a Mexican Hat spatial shape (i.e., excitation from proximal neurons and inhibition from more distal ones) to implement a competitive mechanism. As a consequence, in response to a single input of a given modality, a bubble of neurons is excited within the layer surrounded by an annulus of inhibited neurons. We assumed that lateral synapses are not subject to training.
- A cross-modal input via the cross-modal synapses from neurons in the other sensory modality. Cross-modal synapses are initially set at zero (i.e., there is no multisensory integration before training; this is a reasonable choice, since we do not have any prior information on how visual and auditory stimuli can co-occur, and so no relationship is implemented in the network). Then, these synapses are progressively created during training in presence of a multisensory environment, to incorporate a prior probability on the audio-visual relationship.

The complete set of equations, describing network dynamics, is presented in the Appendix 1 (Supplementary Material).

### Training procedure

The network was trained during a training period, starting from the initial synapse condition described above (large and uniformly distributed RFs, equal for the auditory and the visual nets; cross-modal synapses initially at zero). We used a Hebbian learning rule with a forgetting factor. A synapse is strengthened if the pre-synaptic and the post synaptic activities are high; however, in order to avoid an indiscriminate synapse potentiation, a portion of the previous synapse is lost if the post-synaptic activity is high. This learning rule was adopted, for training both the synapses in the RFs and the cross-modal synapses between the two areas. The equations of synapses training are reported in Appendix 1.

The training procedure consisted of 100 epochs. During each epoch, we presented 360 unisensory visual inputs, 360 unisensory auditory inputs, and 180 cross-modal inputs. Hence, the total number of trial was 90,000, with a ratio “unisensory visual”:“unisensory auditory”:“cross-modal” = 2:2:1. We also performed separate training with different percentages of cross-modal stimuli. The results, not shown for briefness, are briefly commented in the Discussion. A crucial aspect is the definition of possible statistics for the position, the strength and the width of the inputs to be used during training: here, the logic driving the adopted statistics is presented.

#### Strength and width of the inputs: spatial resolution of the stimuli and likelihood functions

In the following, the superscript *S* will be used to discriminate between a visual (*S* = *V*) or an auditory (*S* = *A*) stimulus, reaching the corresponding chain of neurons.

During training, and in the subsequent simulations, we used visual and auditory inputs with a Gaussian shape, centered at an assigned position, θ^*S*^, a standard deviation, σ^*S*^, area, $i_{strength}^{S}$, and superimposed noise, *n*^*S*^. As in the previous paper (Ursino et al., 2017) in order to avoid border effects, we assumed that all distances have a circular shape. In this way, all positions are equal before training, and all observed differences in the azimuthal coordinate are merely a consequence of learning from the environment. As a consequence, we can write the following expression for the spatial distribution of a visual or auditory input as a function of the azimuthal coordinate, ϑ :

(1) $$\begin{array}{l} i^{S}\left(\vartheta \right)=\frac{i_{Strength}^{S}}{\sqrt{2\pi \sigma^{S^{2}}}}\exp\left(-\frac{{\left(d\left(\vartheta^{S},\vartheta \right)\right)}^{2}}{2\sigma^{S^{2}}}\right)\\ \;+n^{S}\left(\vartheta \right)\;S=A\text{ or }V \end{array}$$

where $i_{strength}^{S}$ is the area of the Gaussian function (which can be considered as the strength of the stimulus), θ^*S*^ is the stimulus position (equal to the mean value of the Gaussian function) and *n*^*S*^(ϑ) is a Gaussian white noise term (zero mean value and assigned standard deviation ν^*S*^).

The following equation has been used to compute the circular distance:

(2) $$d\left(\vartheta^{S},\vartheta \right)=\{\begin{array}{lll} \left|\vartheta^{S}-\vartheta \right| & if & \left|\vartheta^{S}-\vartheta \right|\leq 90\\ 180-\left|\vartheta^{S}-\vartheta \right| & if & \left|\vartheta^{S}-\vartheta \right|>90 \end{array}$$

where, 0 < ϑ < 180. According to Equation (2), the position ϑ^*S*^ = 1 deg is equally distant from position ϑ = 180 deg and from position 2 deg, is equally distant from the position 179 deg and from position 3 deg, etc.

It is well-known that the spatial acuity of the visual stimuli is much better in the center (close to the fovea) and progressively deteriorates toward the periphery. In order to simulate a physiological condition, we used an empirical curve from Dacey (1993). This author derived visual acuity from the diameter of the dendritic fields of parvocellular cells: this value (named *D* in the following) is equivalent to the space between two cells. Acuity can be computed as the reciprocal of *D* multiplied by $\sqrt{3}$.

By denoting with θ^*V*^ the position of a visual stimulus in the azimuthal coordinate (i.e., 0 ≤ θ_*v*_ ≤ 180 deg), and with *e*^*V*^ = θ^*V*^−90 the eccentricity with respect to the fovea, the equation from Dacey can be re-written as follows

(3) $$D\left(e^{V}\right)=2.1+0.058e^{V}+0.022e^{V^{2}}-0.00022e^{V^{3}}$$

It is worth noting that the previous equation is expressed in minutes of arc. Hence, to express the same quantity in deg, we need to divide it by 60. Accordingly, visual acuity (in deg) is expressed as follows

(4) $$A\left(e^{V}\right)=\frac{60}{\sqrt{3}D\left(e^{V}\right)}$$

In order to simulate the presence of better acuity at the center, and reduced acuity at the periphery, we assumed that the *SD* of the visual input increases with the eccentricity of the stimulus, following the reciprocal of Equation (4) (i.e., the smaller the acuity, the larger σ^*V*^). We have

(5) $$\begin{array}{l} \sigma^{V}\left(e^{V}\right)=\sigma_{0}^{V}-\frac{\varepsilon }{A(0)}+\frac{\varepsilon }{A(e^{V})}\\ \;=\sigma_{0}^{V}+\frac{\varepsilon \sqrt{3}}{60}\left(D(e^{V})-D(0)\right) \end{array}$$

Equation (5) can be explained as follow: $\sigma_{0}^{V}$ represents the *SD* of the visual inputs at the fovea (i.e., at zero eccentricity). We used the same value as in the previous paper, i.e., $\sigma_{0}^{V}=$ 4 deg. Then, the *SD* linearly increases with the quantity D. Finally, we use a parameter, ε, to adapt the function to the model's capacities. In particular, based on adopted parameter, σ^*V*^ ranges between 4 deg, at 0 eccentricity, to about 12 deg at maximum eccentricity.

The auditory acuity also decreases from the center to the periphery, although it is difficult to quantify this effect being influenced by many factors, such as the stimulus intensity and frequency (Middlebrooks and Green, 1991; Wood and Bizley, 2015). However, this effect is less evident and of smaller entity compared with the visual one (Perrott and Saberi, 1990). Hence, for the sake of simplicity, we assumed that the auditory spatial resolution remains basically constant independently of the azimuthal coordinate. As in the previous paper, we set a value for the auditory *SD* much larger than the visual *SD*: We have σ^*A*^ = 20 deg.

Another important point for the training consists in the choice of the strength for the input (i.e., the quantities $i_{Strength}^{V}$ and $i_{Strength}^{A}$ in Equation 1). These strengths have been chosen so that any unisensory input produces a response, in the corresponding area, close to the maximum saturation. It is worth noting that, due to the presence of a lateral competition, the larger the standard deviation of the input, the greater the input strength required to elicit a consistent response. For this reason, we always used $i_{Strength}^{A}>i_{Strength}^{V}$; moreover, we used a strength of the visual input that moderately increases with the eccentricity. The following empirical law was used for the visual strength as a function of its eccentricity:

(6) $$i_{Strength}^{V}\left(e^{v}\right)=\frac{\sigma^{V}\left(e^{v}\right)}{\sigma_{0}^{V}+\alpha \left(\sigma^{V}\left(e^{v}\right)-\sigma_{0}^{V}\right)}i_{Strength}^{V}\left(0\right)$$

where α is a parameter less than 1. The equation can be explained as follows: the visual strength increases with the standard deviation (provided by Equation 5). However, since a proportional increase produced an excessive activation at the periphery (resulting from our preliminary simulations) this was attenuated by the factor included at the denominator of Equation (6).

Finally, from the previous expressions one can compute the likelihood functions. In particular, by denoting with *I*^*V*^ and *I*^*A*^ the stimuli which reach the network (obtained by sampling Equation 1, i.e., $I^{S}={\left[i_{1}^{S}i_{2}^{S}\cdots i_{j}^{S}\cdots i_{N}^{S}\right]}^{T}$, with *N* = 180, $i_{j}^{S}=i^{S}\left(\vartheta_{j}\right)$ and ϑ_*j*_ = 1, 2, …180), and assuming the independence of noise, we can write

(7) $$p\left(I^{A},I^{V}|\vartheta^{A},\vartheta^{V}\right)=p\left(I^{A}|\vartheta^{A}\right)p\left(I^{V}|\vartheta^{V}\right)$$

where

(8) $$p\left(I^{S}|\vartheta^{S}\right)=\prod_{j\;=\;1}^{N}p\left(i_{j}^{S}|\vartheta^{S}\right)$$

with

(9) $$p\left(i_{j}^{S}|\vartheta^{S}\right)=\frac{1}{\sqrt{2\pi \upsilon^{S^{2}}}}\exp\left\{-\frac{{\left[i_{j}^{S}-\frac{i_{Strength}^{S}}{\sqrt{2\pi \sigma^{S^{2}}}}\exp\left(-{\left(d\left(\vartheta^{S},\vartheta_{j}\right)\right)}^{2}/\left(2\sigma^{S^{2}}\right)\;\right)\right]}^{2}}{2\upsilon^{S^{2}}}\right\}j=1,2,\ldots ,180$$

where in writing Equation (9) we made use of Equation (1). Briefly, the likelihood probability represents the stimulus generative process: Equation (9) implies that the stimulus has a Gaussian shape centered at a given position θ^*S*^, on which a normal Gaussian white noise with zero mean value and standard deviation υ^*S*^ is superimposed. $i_{Strength}^{S}$ represents the area under the stimulus curve (on the average), i.e., the *stimulus strength*, assuming that the higher the area, the higher the effect of the stimulus on the neural net. During training we used $\upsilon^{S}=0.5\frac{i_{Strength}^{S}}{\sqrt{2\pi {\sigma^{S}}^{2}}}$ (i.e., 50% of the maximum input). Different values were used during the testing phase (see Results). Finally, it is worth noting that, in the visual case, the likelihood varies across the visual field due to a change in the parameter σ^*V*^ (see Equation 5) which sets the spatial accuracy of the stimulus, and a parallel change of parameter $i_{Strength}^{V}$ (Equation 6), which sets the stimulus strength.

#### Input positions: probability distribution of the inputs and priors

We assume that the visual input has a greater probability close to the fovea, and smaller probability at the periphery. This corresponds to have a non-uniform prior in visual unisensory conditions. Conversely, since we lack elements to suppose a non-uniform distribution for auditory stimuli, a uniform probability has been used for the auditory unisensory position, as in the previous work. The following probabilities have been used to generate the position of the visual and auditory inputs during training.

##### Visual unisensory prior

The visual position follows a Gaussian distribution, centered at the fovea. Hence

(10) $$p\left(\vartheta^{V}\right)=\frac{1}{\sqrt{2\pi \lambda^{V^{2}}}}\exp\left(-\frac{{\left(\vartheta^{V}-90\right)}^{2}}{2\lambda^{V^{2}}}\right)$$

The standard deviation λ^*V*^ (which here plays the role of a space constant) has been set at 30 deg; i.e., the visual stimuli becomes very rare at ±90 deg eccentricity.

##### Auditory unisensory prior

We maintained a uniform distribution. We have

(11) $$p\left(\vartheta^{A}\right)=\frac{1}{180}$$

##### Cross modal prior

In the cross modal case during training, we assumed that the visual and auditory inputs always originate from proximal spatial positions, i.e., are produced by the same cause. According to the Bayes rule, the joined prior probability can be computed from knowledge of the individual probability of one stimulus, and the conditional probability of the other. A problem is whether, in cross modal conditions, the distribution is dominated by the visual prior (more frequently close to the fovea) or by the auditory one (uniform distribution). We assumed that, in 50% of cases, the cross-modal stimuli follow the visual distribution and in the other 50% of cases follow the auditory one. Hence

(12) $$\begin{array}{l} p\left(\vartheta^{V},\vartheta^{A}\right)=0.5p\left(\vartheta^{V}\right)p\left(\vartheta^{A}|\vartheta^{V}\right)\\ \;+\;0.5p\left(\vartheta^{A}\right)p\left(\vartheta^{V}|\vartheta^{A}\right) \end{array}$$

where we used Equations (10) and (11) for the visual and auditory priors, and the following expression for the conditional probability

(13) $$\begin{array}{l} p\left(\vartheta^{A}|\vartheta^{V}\right)=p\left(\vartheta^{V}|\vartheta^{A}\right)=\beta \frac{1}{180}\\ \;+\left(1-\beta \right)\frac{1}{\sqrt{2\pi \lambda^{{AV}^{2}}}}\exp\left(-\frac{d{\left(\vartheta^{A},\vartheta^{V}\right)}^{2}}{2\lambda^{{AV}^{2}}}\right) \end{array}$$

In writing Equation (13) we assumed that the conditional probability is computed as the weighted sum of a uniform distribution, 1/180, reflecting the moderate possibility that the two stimuli are independent, and a second term, $\frac{1}{\sqrt{2\pi {\lambda^{AV}}^{2}}}\exp\left(-\frac{d{\left(\vartheta^{A},\vartheta^{V}\right)}^{2}}{2{\lambda^{AV}}^{2}}\right)$ reflecting the probability that the auditory and visual events are originated from the same source.

As in the previous work, we used a value of parameter β close to zero and a space constant λ^*AV*^ = 1 deg, assuming that the two stimuli almost always originate from the same source.

### Computation of the estimates

**Figure 2:**
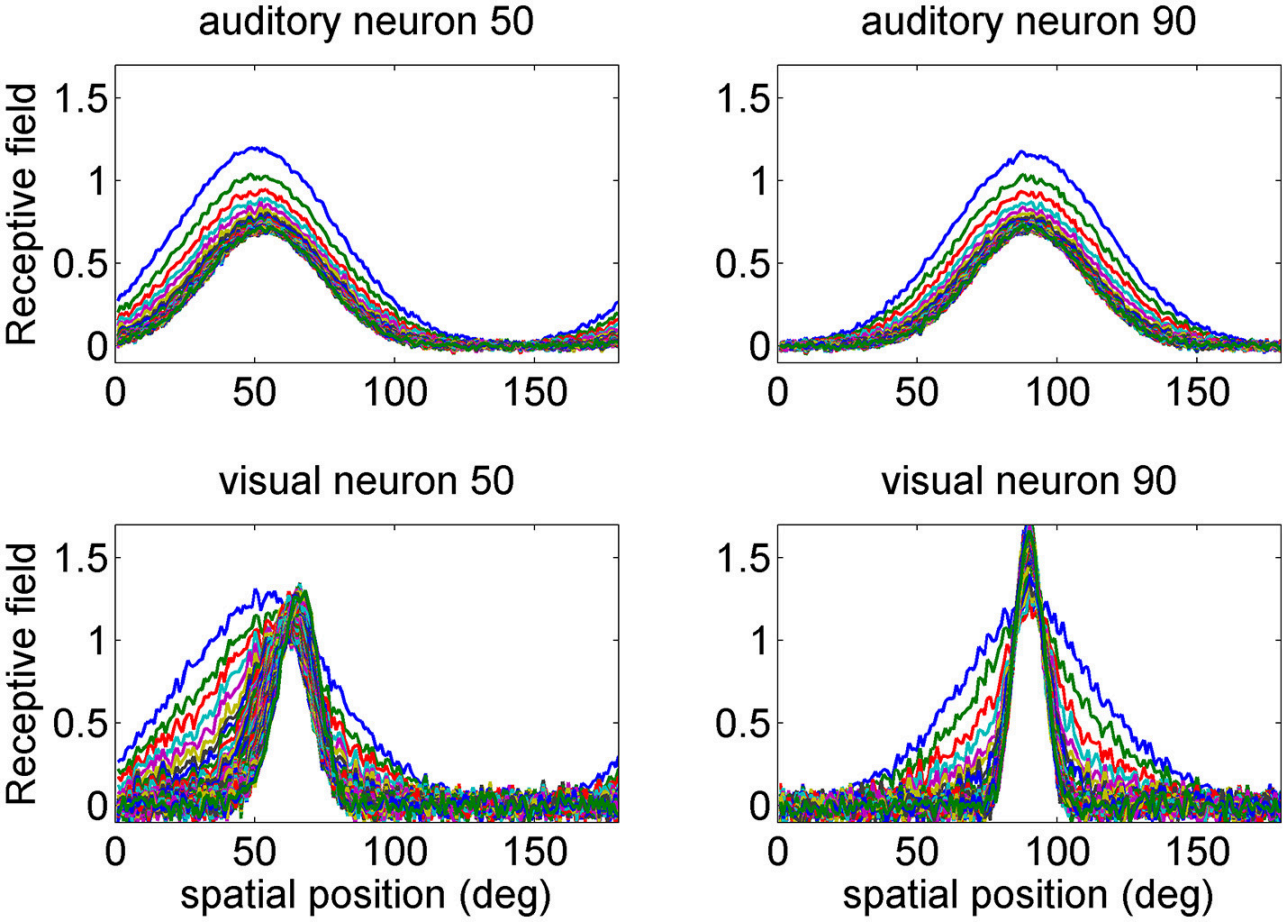
Examples of the progressive shrinking of the receptive fields (RFs) during training. The figures illustrate the RFs of two exemplary neurons in the auditory network (**Upper**) and in the visual network (**Bottom**). The initial preferred positions of these neurons were at 50 and 90 deg (blue lines). It is worth noting that, at the end of training (green lines), the visual RFs are more tuned than the auditory ones, reflecting the more precise spatial localization of the inputs. Moreover, the RF of the visual neuron at initial position 50 deg shifts toward the fovea, as a consequence of the higher prior probability of central visual stimuli. The auditory RFs do not exhibit an appreciable shift.

The preferred position of each neuron after training is calculated using the barycenter of its own RF. However, in order to eliminate the effect of noise (see Figure 2), which produces errors in the computation of the preferred positions, we applied a 0.2 thresholding. Hence, by denoting as $r_{kj}^{S}\text{ with }S=A,V$ the j-th synapse of the receptive field entering a neuron of modality *S* at position *k*, (see the Appendix 1 for the complete equation set), the following expression holds for the neuron preferred position, $\rho_{k}^{S}$

(14) $$\rho_{k}^{S}=\frac{\sum_{j\;=\;1}^{180}{\left[r_{kj}^{S}-0.2\right]}^{+}\vartheta_{j}}{\sum_{j\;=\;1}^{180}{\left[r_{kj}^{S}-0.2\right]}^{+}}\text{ with }S=A,V$$

where, []^+^ is the function “positive part” (i.e., [*y*]^+^ = *y* if y > 0, [*y*]^+^ = 0 if *y* ≤ 0) and ϑ_*j*_ is the position of the input which excites the neuron through the synapse $r_{kj}^{S}$. Since all positions in the model were computed using a circular distance, to avoid border effects (see Equation 2), ϑ_*j*_ was also computed following a circular rule (see the Appendix 2 in the Supplementary Material).

Finally, the network is used to compute the estimated visual and auditory positions, in response to a given noisy unisensory or cross-modal input. These estimates are compared with those provided by a Bayesian estimator with maximal posterior probability.

The auditory and visual positions in the network are estimated by using the barycenter of the activities in the auditory and visual nets, weighted by the preferred positions:

(15) $${\hat{\vartheta }}_{\text{model}}^{S}=\frac{\sum_{k\;=\;1}^{180}y_{k}^{S}{\tilde{\rho }}_{k}^{S}}{\sum_{k\;=\;1}^{180}y_{k}^{S}}\text{ with }S\;=\;A,V$$

where ${\hat{\vartheta }}_{\text{model}}^{A}$ and ${\hat{\vartheta }}_{\text{model}}^{V}$ are the estimated auditory and visual positions, $y_{k}^{A}$ and $y_{k}^{V}$ are the activities of the auditory and visual neurons with label *k* (and preferred position $\rho_{k}^{A}$ and $\rho_{k}^{V}$ respectively). However, in this case too, the preferred positions were re-calculated with a circular shape (say ${\tilde{\rho }}_{k}^{S}$, in the Appendix 2).

Finally, the estimated values have been compared with those obtained from the Bayesian estimator with Maximum posterior probability. The latter is

(16) $$\begin{array}{l} \left[{\hat{\vartheta }}_{Bayes}^{A},{\hat{\vartheta }}_{Bayes}^{V}\right]=\text{arg max }\left\{p\left(\vartheta^{A},\vartheta^{V}|I^{A},I^{V}\right)\right\}\\ \;=\text{arg max }\left\{p\left(\vartheta^{A},\vartheta^{V}\right)p\left(I^{A}|\vartheta^{A}\right)p\left(I^{V}|\vartheta^{V}\right)\right\} \end{array}$$

where we made use of Equations (7–13) to compute the expression (Equation 16).

## Results

### Training of receptive fields

First we analyzed how the receptive fields are affected by training. At the beginning of training, all the receptive fields are large, with the same *SD* (30 deg) both for the auditory and the visual neurons. Moreover, the RFs have an equal spatial distance, i.e., their spatial distribution is uniform. In particular, in this work we used 180 neurons in each area, with an initial preferred direction uniformly distributed from 1 to 180 deg (this signifies that, at the beginning of training, the jth neuron has a RF centered at j deg). During training, the receptive fields progressively shrink, to reflect the *SD* of the input stimuli. Moreover, in the visual case, the preferred direction shifts toward the fovea, due to the greater percentage of central visual stimuli.

Shrinking of the receptive fields is a consequence of the learning rule adopted (a Hebb rule with a forgetting factor, Equation A9 in the Appendix 1). In fact, according to this rule, each receptive field after training becomes equal to its average sensory input (Ursino et al., 2017). Since we assumed that the receptive fields are initially much larger than the inputs, training necessarily results in a progressive reduction of the RF width. In other words, those synapses that are rarely used by the inputs are pruned.

Some examples are presented in Figure 2, where we show the progressive change in the RF for the two auditory neurons with initial preferred position at 50 and 90 deg, and for the two visual neurons with the same initial preferred positions. Two aspects are of value: the visual RFs exhibit a much stronger shrink, which reflects the greater accuracy of the visual stimuli. Moreover, the RF of the visual neuron at preferred position 50 shifts toward the fovea. After training, its preferred position moves at ~65 deg.

In a previous paper (Ursino et al., 2017) we demonstrated that the width of the RFs reflects the likelihood of the inputs. As a new element, the position of the RFs reflects the prior about the frequency of the inputs (in particular, the greater probability to have a visual stimulus close to the fovea, according to Equation 10). As it will be shown below, this prior causes a bias in the visual position estimate in unisensory conditions.

**Figure 3:**
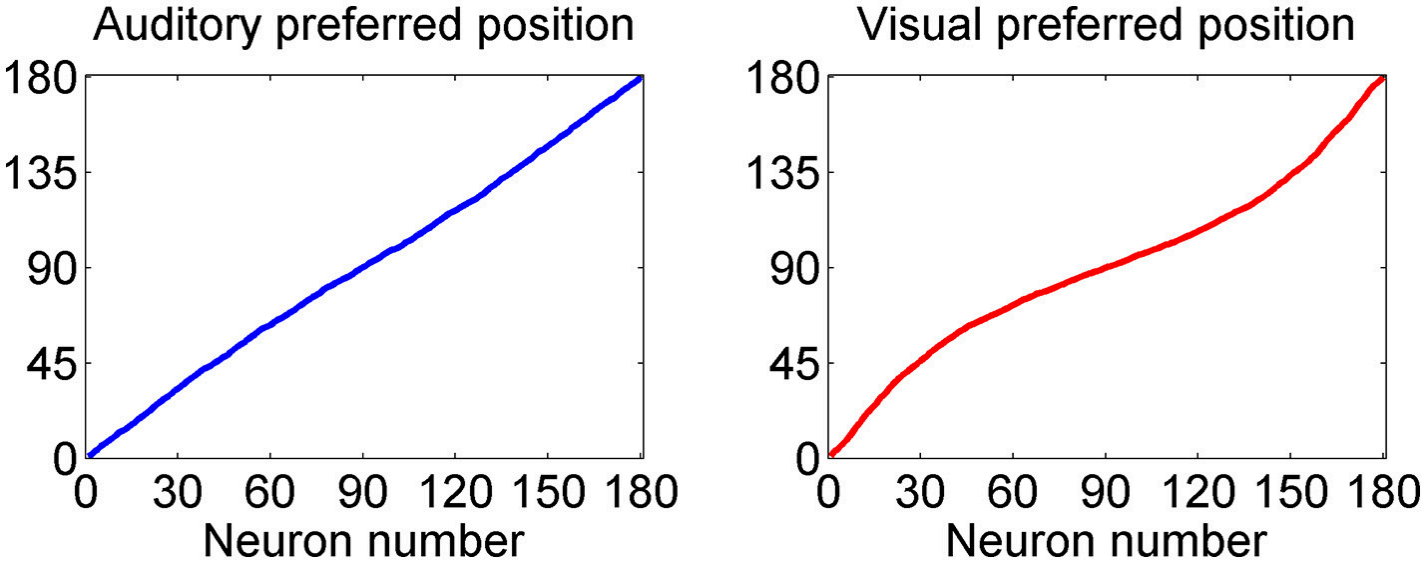
Distribution of the preferred positions for all 180 auditory **(Left)** and visual **(Right)** neurons after training. The distribution of auditory neurons is linear, i.e., the RFs are uniformly distributed, reflecting the uniform unisensory prior. Conversely, the distribution of the visual neurons is denser toward the fovea, reflecting the Gaussian distribution of the prior (with more visual stimuli at the center, and less at the periphery).

This point is further summarized in Figure 3, which describes the preferred positions of all 180 auditory and all 180 visual neurons (computed with Equation 14) after training. As evident from the left panel, the preferred positions of auditory neurons exhibit a uniform distribution; conversely, the preferred positions of visual neurons are thickened around 90 deg: in particular, about 1/3 of visual neurons (i.e., those labeled from 60 to 120) have a preferred position between 72 and 108 deg, and about 1/2 of visual neurons (i.e., those labeled from 45 to 135) have preferred position between 60 and 120 deg.

**Figure 4:**
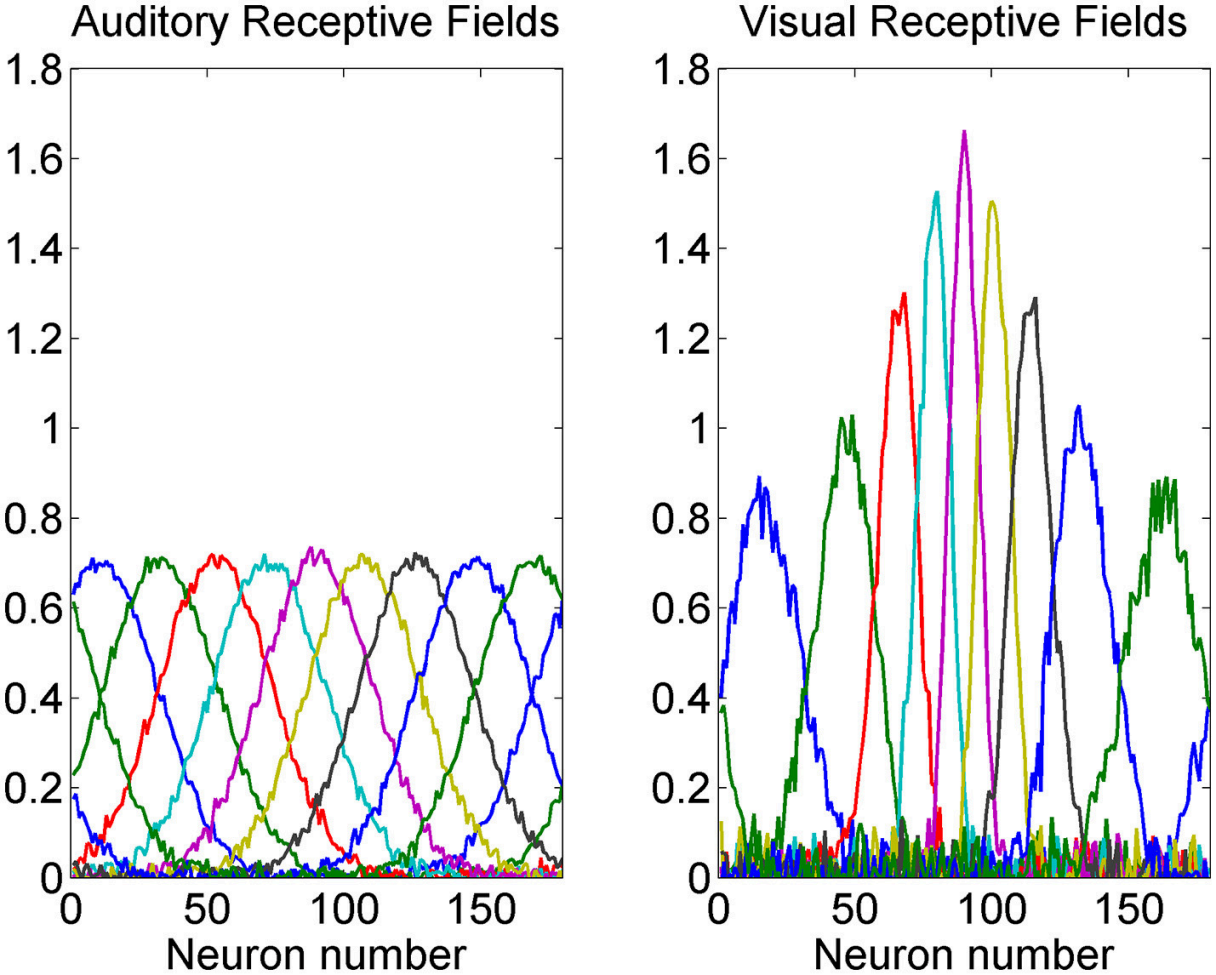
Exempla of auditory **(Left)** and visual **(Right)** RFs after training. We showed the RFs of neurons with initial preferred positions from 10 to 170 deg with a 20 deg step. It is evident that the visual RFs are denser and more precise close to the fovea.

A summary of some auditory and visual receptive fields after training is reported in Figure 4. It is evident the uniform distribution of the auditory RFs, with larger width, and the non-uniform distribution of the visual RFs: they are sharper close to the fovea due to the greater precision of the central visual stimuli, and denser near the fovea, reflecting the prior.

### Training of cross-modal synapses

**Figure 5:**
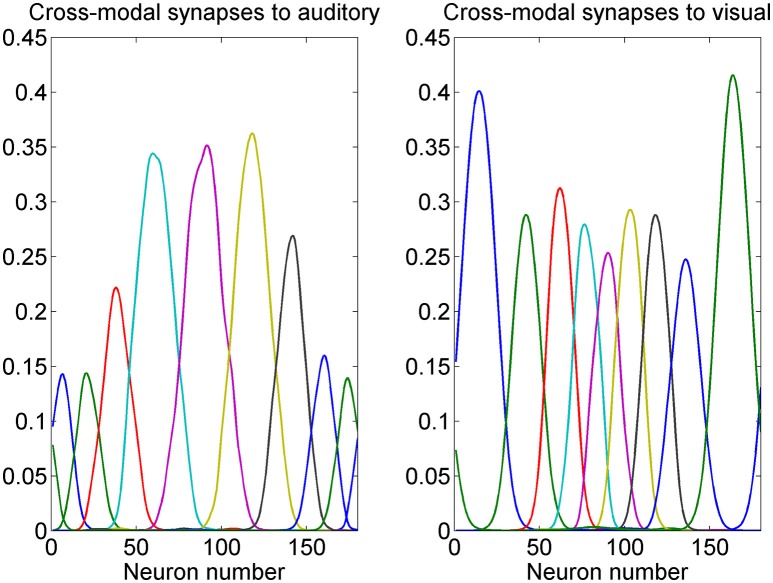
Example of cross-modal synapses after training. Each curve represents the synapses that reach one auditory neuron **(Left)** or one visual neuron **(Right)** from all 180 neurons in the other modality. We showed neurons with initial preferred positions from 10 to 170 deg with a 20 deg step. It is worth noting that auditory neurons receive stronger cross-modal synapses when they are placed toward the fovea, whereas visual neurons receive stronger cross-modal synapses when placed at the periphery. Moreover, each neuron receives synapses only from other neurons with similar preferred positions. These patterns reflect the prior on the proximity of visual and auditory positions in cross-modal stimulation, and the prior on the higher frequency of visual stimuli at the fovea, and scarce frequency of visual stimuli at the periphery.

While the presence of a prior probability of the visual stimuli is reflected in the preferred positions of visual neurons, the prior on the co-occurrence of visual and auditory stimuli is incorporated in the model in the cross modal synapses. The pattern of some cross-modal synapses after training is shown in Figure 5. It is worth noting that these synapses link neurons which have similar preferred positions. In fact, they are created during training thanks to the Hebbian mechanism (see Appendix 1) whenever visual and auditory stimuli occur together. It is worth noting that the visual neurons exert a strong effect on the auditory ones close to the fovea, but have a minor influence at the periphery (since visual stimuli infrequently occur at the periphery). Conversely, the auditory neurons exert quite a uniform effect on the proximal visual ones throughout the azimuthal space, but with major strength at the periphery.

The previous figures describe the effect of training on the RFs and on the cross-modal synapses. Then, we used the trained network to evaluate positions in unisensory and cross-modal conditions, and compare model estimates with those obtained with the Bayesian estimator.

### Spatial position estimate: unisensory stimulation

First, we evaluated model performance in unisensory conditions by assessing the mean value and standard deviation of the estimates at all spatial positions of the input stimuli. The estimates are repeated at different levels of superimposed noise, i.e., using a *SD* of noise (υ^*S*^ in Equation 9) equal to 33, 50, or 66% of the maximum input. Of course, the higher the noise, the higher the standard deviation of the estimates and the higher the effect of prior compared with the likelihood function.

**Figure 6:**
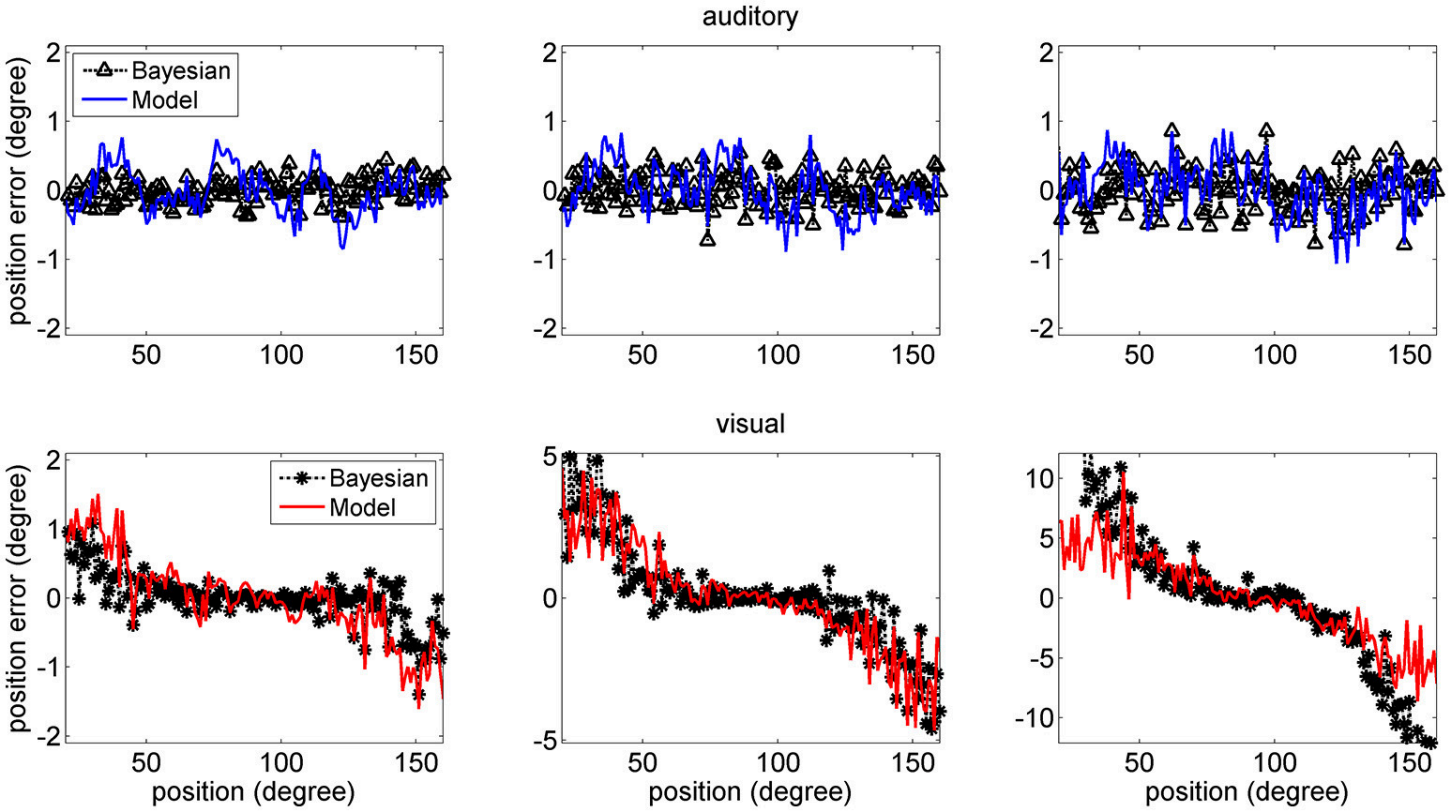
Position errors for the model estimates (Equation 15) for the auditory (**Upper**: blue lines) and visual (**Bottom**: red lines) stimuli in unisensory conditions, as a function of the true stimulus position. Each point is the average of one hundred trials. The left column has been obtained using a *SD* of noise as low as 33% of the maximum input. The middle and right columns have been obtained with a *SD* of noise as high as 50 and 66% of the maximum input, respectively. In these figures, the peripheral space is not shown, due to the large *SD* of the visual estimates (i.e., visual estimates are nor reliable there). It is worth noting the bias of visual estimates toward the fovea, reflecting the non-uniform distribution of the unisensory visual inputs. Moreover, this bias increases with the superimposed noise. Results are compared with those obtained with the Bayesian estimator (Equation 16, black symbols).

The mean values of the position errors (perceived position—real position) are shown in Figure 6 for the auditory (upper panel) and visual (lower panel) cases. However, since the estimates are affected by a large variance at the periphery of the visual field, we focus attention only on the range with acceptable variance. It is worth noting that, for an unbiased estimator, the average position error should be close to zero.

Results show that the mean values of the model estimates substantially agree with those of the Bayesian estimator. In the visual case, we can observe a constant bias: the estimated visual position is significantly shifted toward the fovea (i.e., we have a negative shift at positive eccentricities and vice versa), and this shift is especially evident in the eccentricity ranges ±30–60 deg. This shift increases significantly with the level of superimposed noise (up to about 6–8 deg when noise is as high as 66% of the input) and reflects the effect of the prior information on the visual stimuli. Conversely, the auditory estimates are quite unbiased, i.e., they exhibit almost zero position errors.

**Figure 7:**
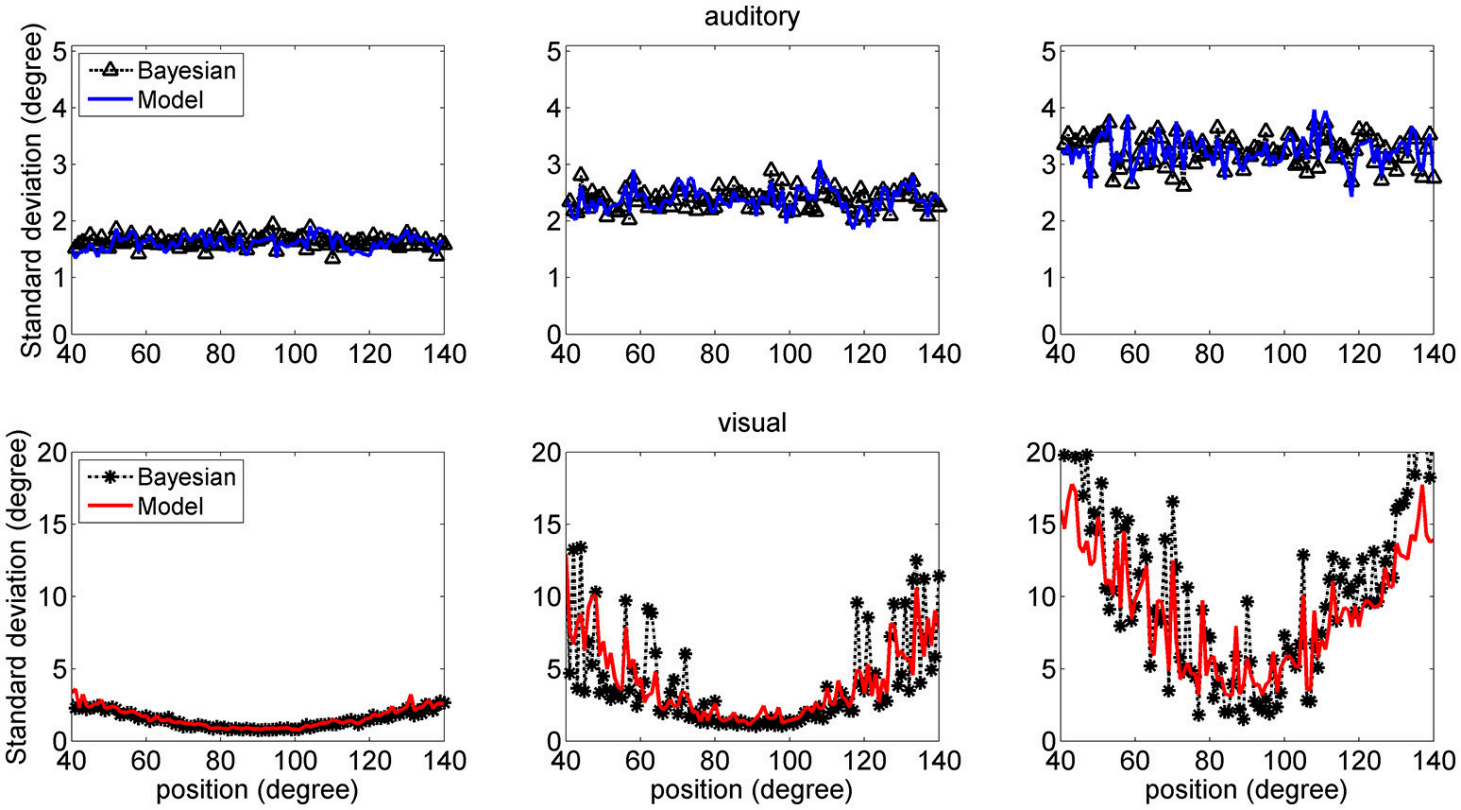
*SD* deviations for the auditory (**Upper**: blue lines) and visual (**Bottom**: red lines) estimates in unisensory conditions, as a function of the true stimulus position. Each point was computed from one hundred trials. Results have been obtained from the same simulation data as in Figure 6. It is worth noting that the *SD* of all estimates increases with the noise level (from left to right, 33, 50, and 66%). Moreover, the visual estimates have smaller *SD* close to the fovea compared with the auditory estimates (0.8 vs. 1.5 deg, left column; 1.2 vs. 2.4 deg middle column; 2 vs. 3.5 deg right colum), but their *SD*s increase at the periphery. Results are compared with those obtained with the Bayesian estimator (Equation 16, black symbols).

Figure 7 shows a comparison between the *SD* of the estimates in the network and in the Bayesian estimator. The agreement is quite good in the overall central range and at all noise levels used. It is evident that the *SD*s of the estimates increase with the noise level (i.e., moving from the left to the right columns in the figure). Moreover, at the fovea the *SD* of the visual estimate is smaller than the *SD* of the acoustic estimate, reflecting the greater accuracy of the visual stimulus. However, the *SD* of the visual estimate increases dramatically at the periphery, as a consequence both of the reduced accuracy of the visual inputs and of the small prior probability.

**Figure 8:**
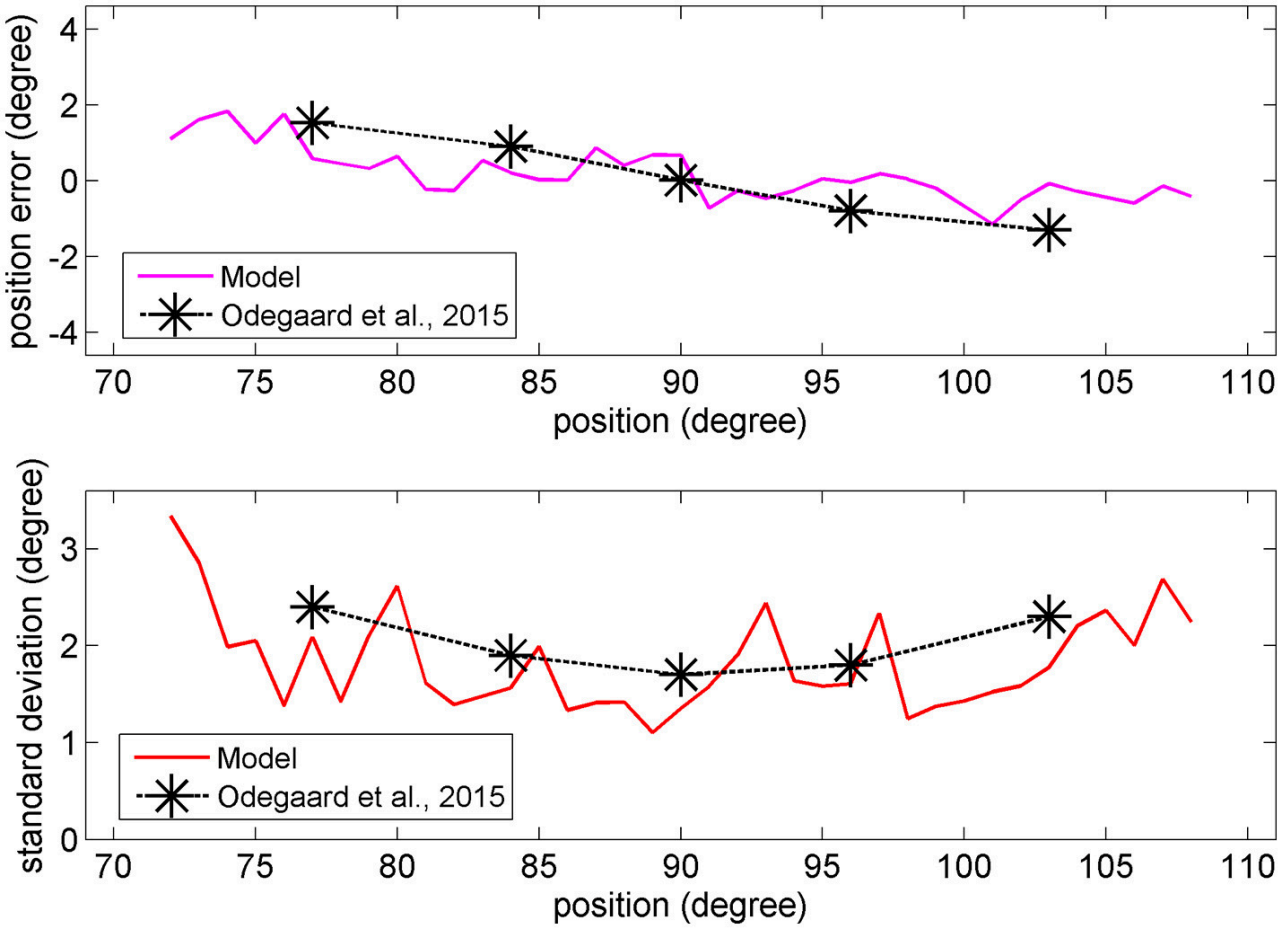
**Upper:** Comparison between the visual estimation bias computed with the model with 66% of superimposed noise (red line), and that reported by Odegaard et al. (2015) (black symbols). **Bottom:** Comparison between the visual *SD* computed with the model with 50% of superimposed noise (red line), and that reported by Odegaard et al. (2015) (black symbols).

In the visual case, results of Figures 6, 7 agree with results by Odegaard et al. (2015). These authors investigated the perception of a visual stimulus vs. the azimuthal coordinate, and observed that this perception is shifted toward the fovea up to about 1.5 deg, if the input is provided in the azimuthal range −13 to +13 deg. Furthermore, the authors observed that the *SD* of the visual estimate moderately increases with the eccentricity in the same azimuthal range. A comparison between model estimates and the visual data by Odegaard et al. (2015) is shown in Figure 8. The present network produces a similar visual shift (i.e., a similar bias of the estimator) (upper panel) and a similar *SD* (bottom panel). Indeed, the standard error of the means in the upper panel seems higher in the model than in the data, but data have been obtained on 412 subjects, who performed 512 trials each, thus strongly reducing the variance of the mean.

Conversely, Odegaard et al. (2015) observed an opposite shift (toward the periphery) for the auditory localization, at variance with the present model. The same observation was also recently confirmed by Garcia et al. (2017). These data are not explained by the model and are further commented in the Discussion.

### Spatial position estimate: cross-modal stimulation

Finally, we performed some additional simulations by providing a cross-modal stimulus.

**Figure 9:**
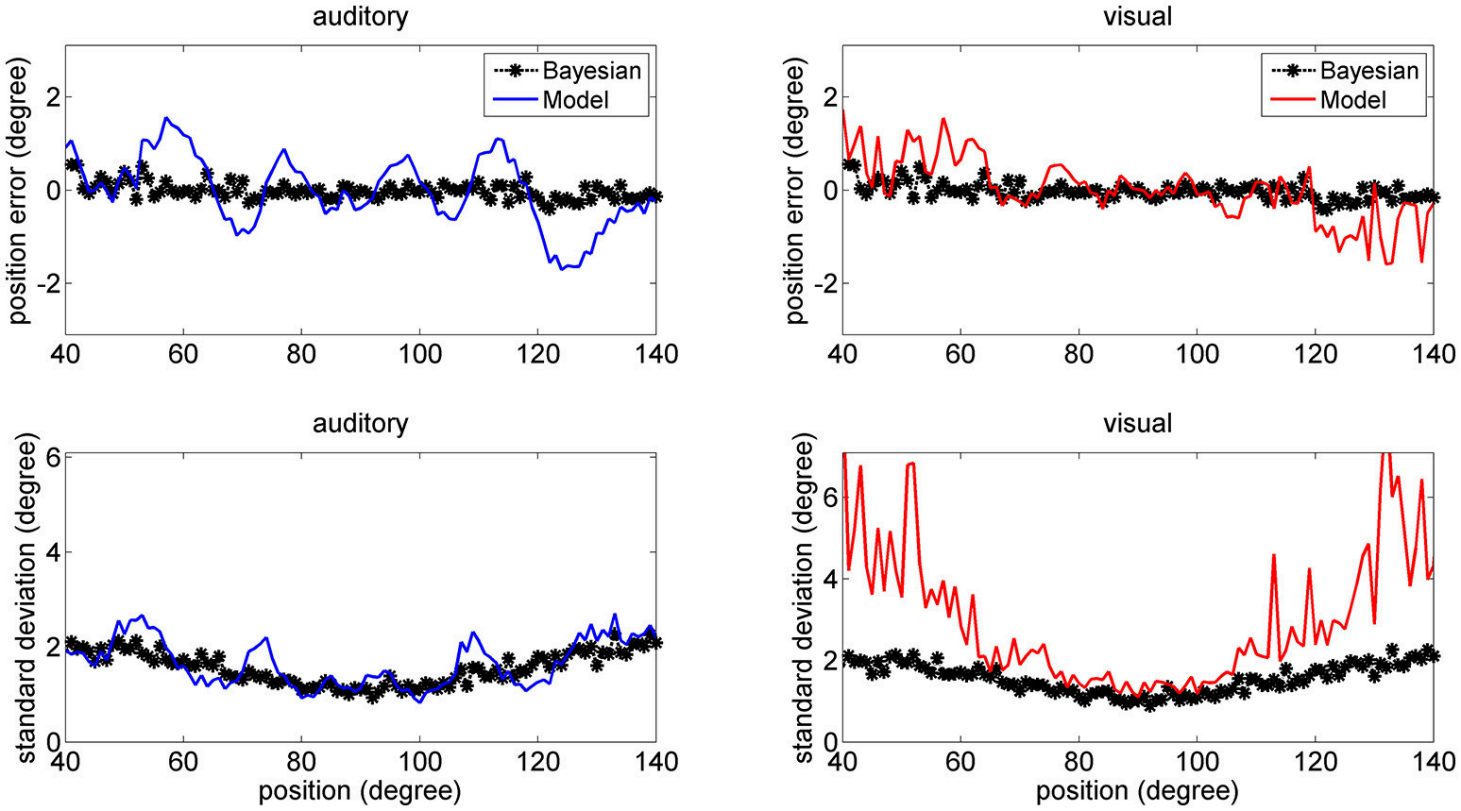
Position errors **(Upper)**, and *SD* of the estimates **(Bottom)** computed with the model (Equation 15) for the auditory (**Left**: blue lines) and visual (**Right**: red lines) stimuli in cross-modal conditions, with the two stimuli at the same position. Each point is the average of one hundred trials. The *SD* of noise was 50% of the maximum input. It is worth noting that the bias of the visual estimate and its *SD* are smaller than in the unisensory case. Moreover, the *SD* of the auditory estimate is significantly smaller than in the unisensory case. Black points are the results of the theoretical Bayesian estimator.

In a first set of trials we provided coincident cross-modal stimuli (100 trials per each position) and evaluated the same quantities as in Figures 6–8. The results are summarized in Figure 9 (for briefness, just the case with 50% noise is shown). Some aspects are of value. For what concerns the visual estimates, first, the bias is significantly reduced compared with the unisensory case due to the presence of a simultaneous auditory stimulus (let us compare the upper right panel in Figure 9, maximum bias = ±2 deg, with the bottom middle panel in Figure 6, maximum bias = ± 5 deg). Moreover, the *SD* of the visual estimate is reduced at the periphery, compared with the unisensory case. Finally, the *SD* of the auditory estimate is also reduced compared with the unisensory case, and in the central range becomes equivalent to the *SD* of the visual estimate.

A significant difference, however, emerges by comparing model predictions with those of the Bayesian estimator. The two estimators provide quite similar results for what concerns the auditory estimate, and are in good agreement for what concerns the visual estimate in the central range (70–110 deg). Conversely, the network visual estimate exhibits a larger shift (but this is just 1 deg) and a larger *SD* (although much smaller than in the unisensory case) compared with the Bayesian one. The reason is that the Bayesian estimator, due to the expression used for the conditional probability (Equation 13) always chooses quite coincident values for the visual and auditory positions. This problem will be analyzed in the Discussion.

In the last set of simulations, we gave the network two cross-modal stimuli at disparate spatial positions, in order to simulate the ventriloquism effect. In particular, the visual stimulus was placed at all positions between 40 and 140 deg, and, at each position, an auditory stimulus was given with a shift in the range −40 to +40 deg (here a positive shift means that the auditory stimulus is located at the left of the visual stimulus, and vice versa). One hundred trials were then repeated per each combination of stimuli, with a 50% noise level and we evaluated the average error in the auditory and visual position estimates.

**Figure 10:**
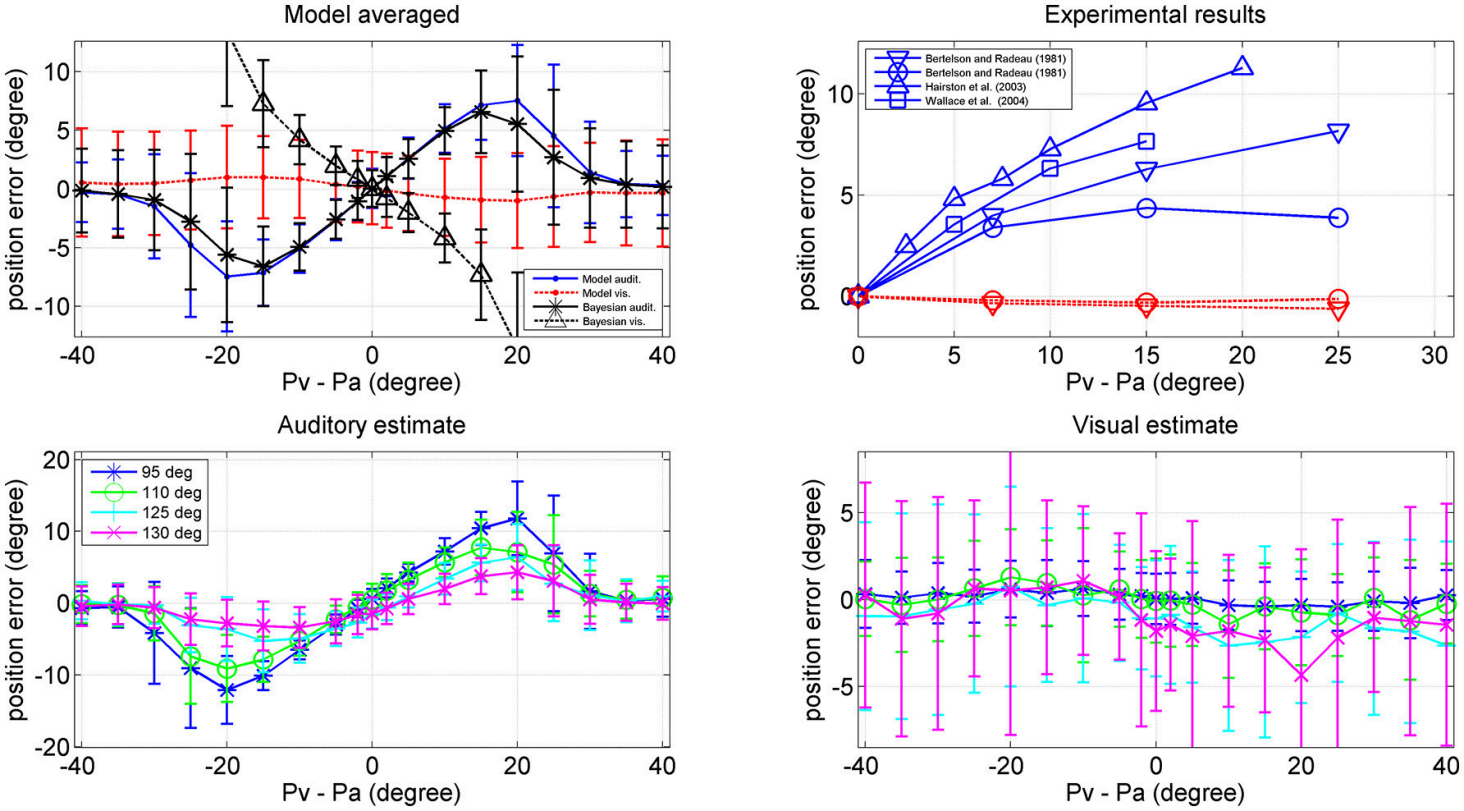
**Upper left:** Ventriloquism effect simulated with the network during cross-modal trials. Cross modal trials were performed, by moving the visual stimulus from position 40 deg to position 140 deg and, at each visual position, adding a second auditory stimulus with a shift in the range from –40 to + 40 deg from the visual one. One hundred trials were performed at each condition, with 50% noise. Results are averaged over all the 100 positions and over all 100 trials per each shift. The x-axis represents the audio-visual distance (where positive values indicate that the visual stimulus is placed on the right), the y-axis is the perceived error (estimated position minus true position): auditory perception, continuous blue line; visual perception, dotted red line. The black lines represent the error of the Bayesian estimate (*auditory, Δ visual) averaged over the same trials. Bars denote standard deviations. **Upper right:** Behavioral data from Hairston et al. (2003) Δ, Wallace et al. (2004) □, Bertelson and Radeau (1981) ∇, and o. **Bottom:** Auditory (Left) and (Right) visual position errors evaluated with the model when the visual stimulus was fixed at position 95 deg (blue), 110 deg (green), 125 deg (cyan), and 130 deg (magenta). The auditory ventriloquism effect decreases with the azimuthal position.

Results are shown in Figure 10. The left upper panel summarizes the results of all trials (i.e., at all positions of the visual stimulus), displaying the perception error (auditory blue, visual red) vs. the audio-visual shift. The auditory perception exhibits a significant bias in the direction of the visual stimulus; this bias increases up to a shift as large as 20–25 deg, and then decreases. The visual perception also exhibits a moderate bias in the direction of the auditory stimulus, but this is quite small (<1 deg). The previous patterns agree with the well-known ventriloquism effect (i.e., a shift of the auditory perception vs. the visual one). By comparison, the rigth upper panel shows some results in the literature (Bertelson and Radeau, 1981; Hairston et al., 2003; Wallace et al., 2004), which confirm a similar trend.

Since an important aspect of this work is the role of the azimuthal coordinate, it is of value to evaluate the dependence of the previous results on the position of the visual stimulus. This is illustrated in the two bottom panels of Figure 10, which summarize the auditory and visual perception errors when the visual stimulus was located at 95, 110, 125, and 130 deg. The auditory ventriloquism effect decreases if the visual input moves from the fovea toward the periphery, in agreement with some data in the literature (Hairston et al., 2003; Charbonneau et al., 2013). Conversely, the mild visual shifts thus not exhibit a significant dependence on the azimuth.

It is worthwhile that the reduction of the ventriloquism effect with the azimuthal coordinate can explain some of the differences among the behavioral data in the right upper panel of Figure 10. Wallace et al. (2004) used random positions for the visual stimulation, including the central position. Bertelson and Radeau (1981) used fixed acoustic stimuli at 10° left or right of the median line and visual stimuli at 7, 15, or 25° at both sides of the acoustic one, thus including also peripheral positions. Hairston et al. (2003), instead, considered different visual position separately (0, 10, and 30°) but maintain the visual target fixed, so is the most similar condition to our simulation. Accordingly, data reported by Hairston et al. for central position exhibit the greatest bias, while those by Bertelson and Radeau the smallest.

**Figure 11:**
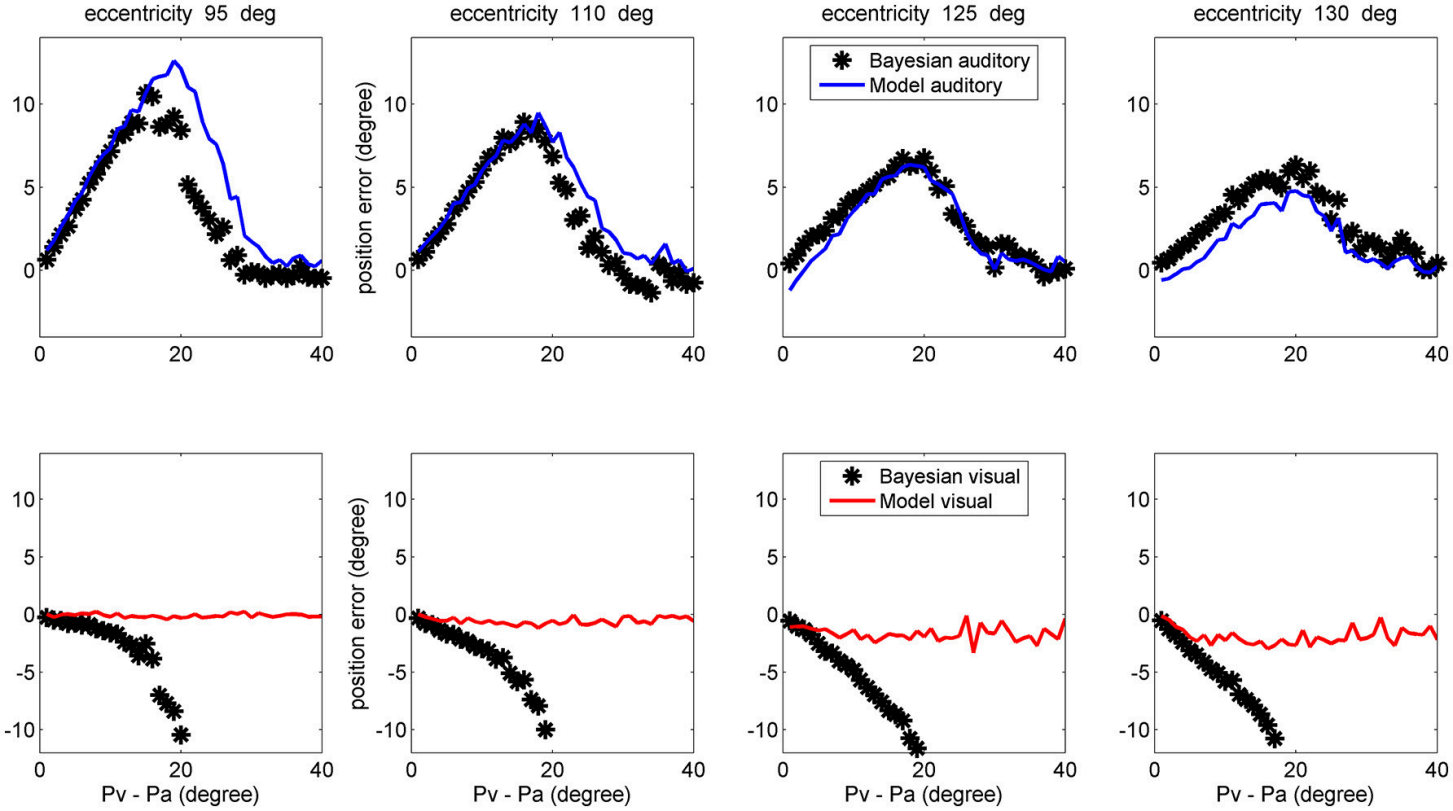
Comparison between the ventriloquism effect simulated with the network and predictions of the Bayesian estimator, evaluated at different azimuthal positions for the visual stimulus (first column 95 deg; second column: 110 deg; third column: 125 deg; fourth column: 130 deg). **Upper:** The auditory bias decreases with the visual azimuthal coordinate, both in the model (blue lines) and in the Bayesian estimator (black). **Bottom:** The model exhibits negligible visual bias (red lines), whereas the Bayesian estimator exhibits a significant visual bias (black) at large audio-visual shifts (visual shifts more negative than 10 deg, occurring at audio-visual disparity lager than 20 deg, are not reported since clearly unrealistic). A 50% noise was used during these trials.

Finally, a comparison between model performance and the Bayesian estimates can be found in the left upper panel of Figure 10 (averaged over all azimuthal positions in the range 40–140 deg) and in Figure 11 (at exemplary positions of the visual stimulus). Several aspects are of value. The pattern of the auditory position bias is similar in the model and in the Bayesian estimator. Both exhibit a linear increase up to a maximum audio-visual shift (~20–25 deg); then the auditory bias decreases to zero at large audio-visual shift. Furthermore, the auditory bias decreases with the azimuthal position of the visual input, although this phenomenon seems more evident in the model than in the theoretical estimator. However, a significant difference is evident between the model and Bayesian visual estimates. The Bayesian estimator predicts a significant visual bias toward the auditory position at large audio-visual shifts (a phenomenon neither produced by the model, nor evident in the behavioral data). The reason for this difference is that the Bayesian estimator tries to maintain the auditory and visual stimuli at very proximal positions. A better Bayesian estimator (more similar to behavioral data) could be designed by distinguishing the case of one causal inference (C = 1) from the case of two distinct causal inferences (C = 2), as done, for instance, by Wozny et al. (2010). This problem will be analyzed in the section Discussion and may be the subject of future work.

## Discussion

The idea that the brain can perform a near-optimal Bayesian inference, thus exploiting multisensory information in an optimal way, has been receiving an increasing interest in the Neuroscience literature. Several recent results confirm that the brain can combine cues from different sensory modalities according to their reliability, in a way quite similar to that performed by a Bayesian estimator, in an effort to maximize the posterior probability of a correct choice (Shams et al., 2000, 2005b; Alais and Burr, 2004; Körding et al., 2007; Gu et al., 2008; Fetsch et al., 2009, 2012; Fischer and Peña, 2011; Cazettes et al., 2016).

Various neuro-computational models (some of which already discussed in Ursino et al., 2014, 2017) analyze how Bayesian estimates can be computed by a population of neurons, assuming that the global population activity can encode the probability distribution. In this regard, Deneve et al. (1999), Ma et al. (2006), and Pouget et al. (2013) demonstrated that a population of neurons can compute the likelihood function of the stimulus, thus realizing an ideal observer. In particular, Deneve et al. (1999) showed that a recurrent network of non-linear units with broad tuning curves can achieve maximum likelihood, provided that the level of noise is independent of firing rate. Assuming a third layer of neurons that encodes the prior probability, Bayesian inference can be realized by simply summing up all population activities (Ma and Rahmati, 2013; Pouget et al., 2013). A population coding approach was also used in a series of studies (Fischer and Peña, 2011; Cazettes et al., 2014, 2016; Rich et al., 2015) to simulate position estimate in the owl's auditory cortex: in a single network of neurons, the authors assumed that the cue repeatability (hence, the likelihood) is represented in the shape of the tuning curves, and that the prior probability is coded in the density of neurons preferred directions.

All previous contributions, however, describe a neuro-computational network at its mature stage, providing suggestions about where the likelihood and prior information may be encoded. None of them describes how a neural network can develop to learn the probabilities (likelihood and prior) under the pressure of external events, starting from an initial immature stage, nor including learning rules for synapse training.

In a previous study (Ursino et al., 2017), we demonstrated, both theoretically and via computer simulations, that a near-optimal Bayesian estimator can develop in a multi-sensory environment using a network consisting of two chains of unisensory neurons (say audio and visual) trained with a realistic learning rule (i.e., a Hebb rule with a forgetting factor). The likelihood functions are encoded in the width of receptive fields, while a prior probability on the co-occurrence of the audio-visual stimuli is encoded in the cross-modal synapses connecting the two areas.

The present study introduces two new elements in the Bayesian scenario, not contemplated in the previous study: first, the accuracy of a stimulus (hence the shape of the likelihood probability) can vary with the azimuthal coordinate. Second, the frequency of the input stimulus may depend on the position too, with some spatial zones more frequently excited than others. This is reflected in a non-uniform unisensory prior. In particular, we assumed that both the accuracy and the probability of visual stimuli are higher near the fovea, and progressively decrease at the periphery of the visual field.

Results confirm that the network can correctly encode these new aspects of the environment, i.e., the non-uniform patterns of the likelihood probability and of the unisensory prior. More in detail:

- the different spatial accuracy of the input is coded in the width of the RF synapses. In fact, during training the RFs progressively shrink (starting from an initial wider configuration) to match the local accuracy of the input (Figure 4);
- the non-uniform spatial prior of the visual stimuli is encoded in the barycenter of the RF synapses, i.e., in the density of the neuron preferred positions. As illustrated in Figure 2, during training the RFs of some neurons shift toward the center of the visual field. As a consequence (Figures 3, 4) a larger density of neurons codes for positions close to the fovea, whereas a sparser population codes peripheral positions. This result agrees with several data from physiology, showing that the magnification factor (i.e., the extent of visual cortex to which a degree of retina projects) reduces by several fold with the eccentricity (Cowey and Rolls, 1974), and with studies that show that early visual experience is fundamental for shaping neural responses (Blakemore and Cooper, 1970; Mitchell et al., 1973). Furthermore, it is worth noting that a similar way to code for the prior probability was proposed by Girshick et al. (2011) and by Cazettes et al. (Fischer and Peña, 2011; Cazettes et al., 2014, 2016; Rich et al., 2015) in a neural network simulating the spatial localization of the owl's auditory system. In the present work, we demonstrated that the same mechanisms exploited by Cazettes et al. (2014, 2016) develop automatically as a consequence of a biologically realistic learning rule. In other words, while in Cazettes et al. the hypotheses were used to build the network, i.e., were a priori incorporated in the network, in the present model they emerge spontaneously after training, from an immature configuration, as a consequence of the assigned statistics of the inputs.

Nevertheless, results in the literature suggest that even visually deprived individuals (such as early blind and anophthalmic patients) exhibit the typical retinotopic structure of V1 (hence a magnification factor) despite retinal input deprivation and absence of visual experience (Bock et al., 2015; Striem-Amit et al., 2015). Of course, this observation does not change the main conclusion of the present study. Here, we simply demonstrated that a prior probability on the unimodal inputs can be acquired from experience, and encoded in the density of the receptive fields. Consequently, a near-optimal Bayesian estimate can be achieved in case of a non-uniform unimodal prior too. Of course, it is possible that part of this prior is already encoded and present at birth.

The higher density of preferred positions close to the fovea has important consequences for the perception of a single unisensory input. In unisensory conditions, visual estimates exhibit a significant bias toward the fovea (see Figure 6), which is a direct consequence of a denser neuron distribution and a greater accuracy at the center. This bias augments with the level of superimposed noise, when the likelihood functions become less accurate, and so the estimator places more weight on the prior, and is reflected in a high *SD* of visual estimate at high eccentricity, as evident in Figure 7. This model prediction matches behavioral data (Odegaard et al., 2015) and essentially agrees with the prediction of a Bayesian estimator based on the same probabilities (Figures 6, 7).

- (iii) Besides the previous two aspects, a third information is encoded in the network in the form of cross model synapses linking the two areas. As analyzed in the previous work (Ursino et al., 2017) these synapses encode the prior information on the conditional probability.

Results of the model in cross-modal conditions agree with several behavioral data. As evident in Figure 9, in case of coincident stimuli, the *SD* of the estimates is reduced in cross-modal conditions compared with that computed in analogous unisensory conditions. This result underlines the advantage of multisensory integration, especially in conditions characterized by a large level of inaccuracy (such as in case of a single unisensory auditory cue, or an eccentric isolated visual cue).

Some illusory phenomena (not only the ventriloquism, but also the fission effect, in which two auditory beeps modify the perception of a visual flash, Shams et al., 2000, 2005a; Cuppini et al., 2014) can be explained by these synapses developments. Furthermore, in the previous paper (Ursino et al., 2017) we showed that the model, with addition of a third layer of multisensory neurons, can also explain the results by Alais and Burr (2004), concerning bimodal localization of a single event after manipulation of the visual input.

A new aspect, however, is evident in Figure 5 compared with our previous paper. Cross-modal synapses are not only affected by the conditional probability (i.e., Equation 13) but also by the unisensory prior (Equation 10). In fact, the auditory neurons receive stronger cross-modal synapses close to the fovea, where the density and accuracy of the visual stimuli is higher. Therefore, ventriloquism is higher at the center. Conversely, visual neurons receive stronger cross-modal synapses at the periphery: here, isolated visual stimuli are quite infrequent, but a visual cue can be reinforced by the presence of a simultaneous peripheral auditory cue.

The observed dependence of the ventriloquism on the azimuthal coordinate agrees with some behavioral data, although just a few studies examined this point. Hairston et al. (2003) refer that bias declines with target eccentricity (see, for instance, Figure 2 in their paper). The authors then conclude that “central visual stimuli had a substantially greater biasing effect on auditory target localization than did more peripheral visual stimuli”. A similar result is reported by Charbonneau et al. (2013). It is worth noting that this progressive decrease in the auditory bias also agrees with the prediction of the Bayesian estimator, as evident in the upper panels of Figure 11. Finally, we can observe that the decrease in the ventriloquism effect with eccentricity can at least in part explain the differences between the results by Hairston et al. (2003), Wallace et al. (2004), and Bertelson and Radeau (1981), as previously commented in section results.

It is worth noting that, during the present training, we used a percentage of cross-modal inputs as low as 20% of total. We also performed some trials by modifying this ratio (for instance, by using 30 or 40% of cross-modal stimuli). The results (not reported here for briefness) indicate that a larger percentage of congruent cross-modal inputs produces stronger cross-modal synapses, and so a greater ventriloquism effect (for instance, an auditory perceptual bias as large as 12–14 deg).

Lastly, we wish to comment on some limitations of the present model and on the similarities/differences between model predictions and those of the maximum posterior probability estimator.

For what concerns a comparison between the model and the Bayesian estimates, we found a satisfactory agreement in unisensory conditions, both for what concerns the bias (Figure 6) and *SD* (Figure 7) at different noise levels. Some differences can be found, for what concerns the unisensory visual estimates, only at the periphery (i.e., at an eccentricity higher than 60–70 deg) where, however, the frequency of the visual stimuli becomes too low to produce reliable predictions.

Furthermore, a very good agreement can be found for what concerns the auditory estimate in cross modal conditions (Figures 9, 11) both when congruent and incongruent audio-visual inputs are used. Conversely, we noticed some severe differences between model visual estimate and the Bayesian visual estimate in cross modal conditions. These differences are evident when using congruent audio-visual stimuli at an eccentricity greater than 30 deg (Figure 9 right panels). Nevertheless, studies that refer similarities between human behavior and Bayesian estimate rarely consider such levels of eccentricity. Large differences are also evident when using incongruent audio-visual stimuli (Figure 10 left-upper panel, Figure 11 bottom panels). In the latter case (i.e., during ventriloquism), the Bayesian estimator predicts a large visual shift which, however, is not observed in the behavioral data.

There are various possible explanations for these differences. First, in the expression of the Bayesian estimator we used the exact equations for the prior and conditional probabilities. Conversely, a real Bayesian estimator should be constructed from the actual data, i.e., using expressions for the probabilities estimated from experiments. This would increase the variance of the Bayesian estimator compared with the purely theoretical formulas. Second, during training we always used congruent cross-modal stimuli, i.e., we assumed a common cause. This is equivalent as considering that a separate stage identifies congruent stimulus pairs, and that this information is used for training the circuit. As a consequence, we used a very small value for parameter β in Equation (13). This signifies that the Bayesian estimator cannot correctly manage the case of incongruent cross-modal stimuli and, in case of too distant audio-visual inputs, it tries to move also the visual estimate in the direction of the auditory one (lower panels of Figure 11). A more reliable expression for the Bayesian estimator should be constructed by separately considering the case of one single cause (C = 1) and two separate causes (C = 2), as in Wozny et al. (2010), and by repeating the training procedure including both possibilities.

It is worth noting that in the present model, as in the previous one (Ursino et al., 2017) we used cyclic boundary conditions. These have been utilized to avoid a consistent bias from the extreme periphery to the center, induced by the absence of a tail in the sensory input. Indeed, with the use of a cyclic boundary, all spatial positions have potentially the same capacity to deal with sensory inputs, and differences emerge only from experience. We are aware that cyclic boundary conditions are not physiological. Hence, we repeated all simulations without them. Results (not published for briefness) remain essentially the same as in the present work in the range 20–160 deg for what concerns the visual inputs, and in the range 40–140 deg for the auditory and cross-modal ones (since the auditory stimuli have a wider spatial extension). In particular, we claim that the observed differences between the model and the Bayesian estimates (Figures 9–11) cannot be ascribed to the cyclic boundary.

As a last point, we stress that a limitation of our model, which may be the target of future studies, concerns the description of the auditory net. While the visual net can be considered a good replication of the primary visual areas, where a spatial topological organization is already present, the primary auditory cortex is not spatially organized, and spatial information on the auditory stimuli is extracted only at higher stages of the auditory pathway, from interaural time difference or interaural phase difference (Saberi et al., 1998; Recanzone and Sutter, 2008). Although the basic idea of this work (i.e., that cross modal synapses are created linking elements of the visual and auditory nets participating to the same task, or which code for similar events), has probably a general validity to implement conditional priors (see Ursino et al., 2015; Zhang et al., 2016 for the application of similar ideas in a wider context) a more physiological description of the auditory processing stage is needed in future model developments.

In this regard, we stress that some behavioral data (Odegaard et al., 2015; Garcia et al., 2017) show that the auditory localization estimate, in unisensory conditions, exhibits a bias toward the periphery, i.e., auditory unisensory cues are perceived as more eccentric than they are. The present model cannot reproduce this observation. This bias, however, is not reported in all studies and seems significantly affected by the experimental conditions, as shown in Lewald et al. (2000). A more sophisticate auditory network will be the subject of future work, to improve the neurophysiology of this model and unmask possible additional, still unknown mechanisms.

## Authors contributions

MU contributed to the conception and design of the work, carried out the computational model, contributed to the interpretation of results, and drafting of the manuscript. AC contributed to the conception and design of the work, carried out the simulation model, and contributed to the interpretation of results and to a critical revision of the work. GdP contributed to a critical revision of the work and to the interpretation of the results. EM and CC contributed to the conception and design of the work, to the interpretation of results and to a critical revision of the work.

### Conflict of interest statement

The authors declare that the research was conducted in the absence of any commercial or financial relationships that could be construed as a potential conflict of interest.
