# Supplementary material for: Development of a Bayesian Estimator for Audio-Visual Integration: A Neurocomputational Study
Source: Front Comput Neurosci. 2017 Oct 4;11:89. doi: 10.3389/fncom.2017.00089 (PMC5633019; doi:10.3389/fncom.2017.00089)
Supplement: Supplementary file 1 [file Presentation1.pdf]

Supplementary Material – Appendix 1 and Appendix 2

" Development of a Bayesian Estimator for Audio-Visual Integration: A Neurocomputational Study" by M. Ursino et al.

Appendix 1: Network Equations

Network dynamics

The neural network model consists of two chains of N unisensory neurons (Fig. 1). Each neuron codes for a particular spatial position in its modality. Moreover, each chain is topologically organized, i.e., proximal neurons code for proximal positions. In the following, we will denote with a superscript the particular area (auditory or visual) and with a subscript the neuron position within the area.

Each neuron receives three different kinds of inputs: a sensory input from the environment (say u), a lateral input from neurons of the same modality (say l) and a cross-modal input from neurons of the other modality (say c). The global input (equal to the sum of the previous three contributions) is then passed through a sigmoidal relationship, $\varphi(\;)$, which accounts for the presence of a lower threshold and upper saturation in neuron activity, and a first-order low-pass filter with time constant τ, which accounts for the neuron integrative capacity.

Hence, for the generic k-th neuron in the modality S (S = A or V for the auditory and visual modalities, respectively) we can write

$$\tau \frac{dy_k^S}{dt} = -y_k^S + \phi\left(u_k^S + l_k^S + c_k^S\right) \tag{A1}$$

where y_k^S represents the neuron output, and the sigmoidal relationship is described by the following equation

$$\phi(x) = \frac{1}{1+\exp(-s(x-x_0))} \tag{A2}$$

s and x_0 are parameters, which set the slope and the position of the sigmoidal relationship. According to Eq. (A2), the neuron output activity is normalized between 0 and 1 (zero means a silent neuron, one a maximally activated neuron).

It is worth noting that, for the sake of simplicity, we used the same parameters (τ, s and x_0) for all neurons independently of their modality. This choice was adopted to minimize the number of model assumptions.

The expression for the sensory input is computed as the scalar product of the sensory representation of the stimulus ($I^S = [i_1^S\, i_2^S \cdots i_j^S \cdots i_N^S]^T$ see Eqs. (1) − (6)) and the neuron receptive field ($R_k^S = [r_{k1}^S\, r_{k2}^S \cdots r_{kj}^S \cdots r_{kN}^S]^T$) :

$$u_k^S = \sum_{j=1}^{N} r_{kj}^S i_j^S \tag{A3}$$

We assumed that the neuron receptive field, R_k^S, has initially a large extension, described with a Gaussian function, and then progressively shrinks during training, to fit the width of the external input.

The lateral input is computed as follows

$$l_k^S = \sum_{j=1}^{N} \lambda_{kj} y_j^S \tag{A4}$$

where λ_{kj} represents a lateral intra-area synapse connecting the presynaptic neuron j to the post synaptic neuron k in the same area. Here we used the classical Mexican-hat arrangement: a neuron is excited by proximal neurons in the same area, and inhibited by more distal ones

$$\lambda_{kj} = \lambda_{ex} \exp\left(-\frac{d(\vartheta_j, \vartheta_k)^2}{2\sigma_{ex}^2} \right) - \lambda_{in} \exp\left(-\frac{d(\vartheta_j, \vartheta_k)^2}{2\sigma_{in}^2} \right) \tag{A5}$$

where $\lambda_{ex}, \lambda_{in}, \sigma_{ex}, \sigma_{in}$ are parameters which set the strength and width of the excitatory and inhibitory portions of the Mexican hat. In particular, we have $\lambda_{ex} > \lambda_{in}$ and $\sigma_{ex} < \sigma_{in}$. Moreover, $d(\vartheta_j, \vartheta_k)$ represents the distance between neurons' preferred positions, i.e.

$$d(\vartheta_j, \vartheta_k) = \begin{cases} |\vartheta_j - \vartheta_k| & if \; |\vartheta_j - \vartheta_k| \le 90 \\ 180 - |\vartheta_j - \vartheta_k| & if \; |\vartheta_j - \vartheta_k| > 90 \end{cases}. \tag{A6}$$

It is worth noting that we used the same expression of lateral synapses (Eq. A5) in both the auditory and visual areas, to limit the number of model assumptions.

Finally, the cross-modal term in Eq. (A1) is computed as the convolution of the vector of cross modal synapses and the activity in the other unisensory area, i.e.

$$c_k^S = \sum_{j=1}^{N} w_{kj}^{SQ} y_j^Q \qquad \text{with } S = A \text{ or } V \quad Q = A \text{ or } V \text{ with } S \ne Q \tag{A7}$$

where w_{kj}^{SQ} represents a cross-modal synapse from the pre-synaptic neuron j in the area Q to the post-synaptic neuron k in the area S. We assumed that the cross-modal synapses are initially ineffective and are progressively reinforced during the training phase.

Training the network

Starting from the initial basal value of synapses, the network has been trained during a training period in which the sensory input representations (i.e., I^A and I^V) have been given with a random distribution. The shape of the inputs, their strength, superimposed noise and random positions (in unisensory and cross-modal conditions) have been described in the text (see Eqs. (1) – (13)). During training we assumed that the standard deviation of noise (say υ^S in Eq. (1) (9)) is a portion of the maximum input. Hence

$$\upsilon^S = \eta \frac{i_{Strength}^S}{\sqrt{2\pi\sigma^{S2}}} \qquad S = A, V \tag{A8}$$

In the present simulations we assumed η = 0.5 during training and η = 0.33, 0.5 or 0.66 in evaluating network performances after training.

The synapses describing the receptive field, r_{kj}^S ,and those describing the cross-modal link between the two areas, w_{kj}^{SQ}, have been trained using a learning rule with a classical Hebbian potentiation factor and a decay term. We can write, in scalar form

$$\Delta r_{kj}^S = \gamma \, y_k^S \left(i_j^S - r_{kj}^S \right) \quad \text{with } S = A,\ V \tag{A9}$$

$$\Delta w_{kj}^{SQ} = \gamma \, y_k^S \left(y_j^Q - w_{kj}^{SQ} \right) \quad \text{with } S = A,V \quad Q = A,V \quad Q \neq S \tag{A10}$$

Eqs. (A9) and (A10) have been applied, at each step, using the final steady state values of the neuron output (i.e., when transient phenomena are exhausted).

At the beginning of training all cross-modal synapses are assumed equal to zero. Conversely, the receptive-field synapses have a broad spatial extension, and moderate amplitude, identical for the two modalities, i.e.

$$r_{kj}^S = r_0 \exp\left(-\frac{\left(d\left(\vartheta_j, \vartheta_k\right)\right)^2}{2\sigma_R^{\;2}} \right) \quad \text{with } S = A,V \tag{A11}$$

where r_0 sets the initial strength of the receptive field, and σ_R establishes its initial spatial extension (we assume $\sigma_R > \sigma_A$ and $\sigma_R > \sigma_V$ i.e. a wide initial receptive field) . Of course, Eq. (A11) holds only at the first step of training.

A list of all model parameters is given in Table 1.

Table 1

Parameters values

$i_{Strength}^A = 36$	$i_{Strength}^V = 20$	$\sigma^A = 20\,\text{deg}$	$\sigma_0^V = 4\,\text{deg}$	$\varepsilon = 7$	$\alpha = 0.78$
$\eta = 0.5$ (training)	$\eta = 0.33, 0.5, 0.66$ (testing)	$\lambda^V = 30\ deg$	$\lambda^{AV} = 1\ deg$	$x_0 = 0.7$	$s = 0.7$
$\tau = 5$ ms	$\lambda_{ex} = 1.9$	$\lambda_{in} = 1.85$	$\sigma_{ex} = 12$ deg	$\sigma_{in} = 24$ deg	$r_0 = 1.5$
$\sigma_R = 30\,\text{deg}$	$\gamma = 0.04$	$\beta = 10^{-16}$			

Appendix 2

Circular rule used to compute the input positions in Eq. (14)

$$\text{if } k < 90 \quad \text{then} \quad \vartheta_j = \begin{cases} j & \text{if } 1 \le j \le 90 + k \\ j\text{-}180 & \text{if } 90 + k < j \le 180 \end{cases}$$

$$\text{if } k = 90 \quad \text{then} \quad \vartheta_j = j$$

$$\text{if } k > 90 \quad \text{then} \quad \vartheta_j = \begin{cases} j + 180 & \text{if } 1 \le j < k - 90 \\ j & \text{if } k\text{-}90 \le j \le 180 \end{cases}$$

Circular rule used to compute the preferred positions in Eq. (15)

Let us assume that a stimulus of modality S was given at position ϑ^S (and so, the activity in the network of the same modality is approximately centered at that position). We have

$$\text{if } \vartheta^S < 90 \quad \text{then} \quad \tilde{\rho}_k^S = \begin{cases} \rho_k^S & \text{if } 1 \le \rho_k^S \le 90 + \vartheta^S \\ \rho_k^S\text{-}180 & \text{if } 90 + \vartheta^S < \rho_k^S \le 180 \end{cases}$$

$$\text{if } \vartheta^S = 90 \quad \text{then} \quad \tilde{\rho}_k^S = \rho_k^S$$

$$\text{if } \vartheta^S > 90 \quad \text{then} \quad \tilde{\rho}_k^S = \begin{cases} \rho_k^S + 180 & \text{if } 1 \le \rho_k^S \le \vartheta^S - 90 \\ \rho_k^S & \text{if } \vartheta^S - 90 < \rho_k^S \le 180 \end{cases}$$
